# Lipocalin2 Protects Human Embryonic Kidney Cells against Cisplatin–Induced Genotoxicity

**Published:** 2018

**Authors:** Fatemeh Sadeghi, Mahmoud Etebari, Mehryar Habibi Roudkenar, Ali Jahanian-Najafabadi

**Affiliations:** a *Student Research Center, School of Pharmacy and Pharmaceutical Sciences, Isfahan University of Medical Sciences and Health Services, Isfahan, Iran.*; b *Department of Toxicology and Pharmacology, School of Pharmacy and Pharmaceutical Sciences, Isfahan University of Medical Sciences and Health Services, Isfahan, Iran. *; c *Medical Biotechnology Research Center, Paramedicine Faculty, Guilan University of Medical Sciences, Rasht, Iran. *; d *Department of Pharmaceutical Biotechnology, School of Pharmacy and Pharmaceutical Sciences, Isfahan University of Medical Sciences and Health Services, Isfahan, Iran.*

**Keywords:** Cisplatin, Lcn2, Oxidative stress, Genotoxicity

## Abstract

Cisplatin is one of the most useful chemotherapeutics which performs its cytotoxic effect via accumulation of platinum resulting in oxidative stress, and destruction of cell DNA. This could probably cause secondary cancers in healthy tissues. Lipocalin2 (Lcn2) is a protein which its expression is increased in oxidative stresses. Therefore, the present study was performed to evaluate the protective effects of Lcn2 up-regulation on cisplatin genotoxicity. In order to up-regulate Lcn2 expression, HEK293 cells were transfected with pcDNA3.1-Lcn2 vector. Afterwards, stable cells consistently expressing Lcn2 were selected via screening with G418 antibiotic. Next, overexpression of Lcn2 was evaluated by RT-PCR and ELISA, comparing to the control non-transfected cells. Then, in order to evaluate the cytoprotective effects of Lcn2 overexpression, transfected and non-transfected cells were subjected to cisplatin treatment followed by MTT and alkaline Comet assays. RT-PCR and ELISA assays confirmed up-regulation of Lcn2 by the stable cells. MTT assay of the Lcn2 over-expressing cells showed higher IC50 values comparing to the non-transfected cells. Furthermore, the Comet assay confirmed Lcn2 protective effects on the cisplatin (1 µg/mL) induced genotoxicity. In the present study, for the first time, we showed the protective effect of Lcn2 on cisplatin induced genotoxicity. Therefore, one of the probable mechanisms of Lcn2 cytoprotctive effects under oxidative stress conditions could be due to the prevention of genotoxicity. However, further evaluations in this regard must be considered.

## Introduction

Cancer is one of the main health issues in almost all countries. For example, in the United States one of every four deaths is due to cancer. Three main strategies in treatment of cancer are surgery, radiotherapy, and chemotherapy (1). Most chemotherapeutics impose oxidative stresses on cancerous cells which finally results in their death (2). Oxidative damage is a well-known mechanism in cell and tissue damage which is caused by free radicals and active oxygen species (ROS) (3, 4). Free radicals can pair with cell components and destroy proteins and nucleic acids. However, off target effects of chemotherapeutics, *i.e.* affecting non-cancerous cells, could result in acute and chronic side effects including malfunction of heart, nervous system, and liver. Platinum derived chemotherapeutics, headed by cisplatin, is one of the most widely used treatments for various types of cancer (5). Cisplatin,(cis-diammine-dichoro-platinum) also known as CDDP, is a synthetic, anti-tumor compound that is commonly used in treatment of malignant tumors of ovary, lungs, head and neck, and testicles (6-8). It conducts its cytotoxic effects via accumulation of platinum in cells and triggering oxidative stresses (5, 9). In addition, it binds to cellular DNA and prevents replication and mitosis. Although cisplatin is more effective on cells with high proliferation rate, specifically cancerous cells, but, it also affects cells with lower proliferation rates (5, 10). This non-specific activity would result in serious side effects including secondary cancers (11). Lipocalin2 belongs to a very large, varying family of soluble, often secreted proteins of lipocalins which are capable of performing various actions including transferring retinols and pheromones, synthesizing prostaglandins and modifying immune system responses, and plays role in cellular hemostasis (12, 13). It is known as a secreted protein in epithelial cells, macrophages, and neutrophils (14, 15). Naturally, Lcn2 is expressed at low concentrations in some human tissues including bone marrow (16), tubules of kidneys, trachea, lungs, stomach, and large intestine. Skin inflammation (17) and some types of cancer can increase the concentration of Lcn2 in the mentioned tissues (18). 

Different studies showed that Lcn2 has a protective effect against toxicity caused by H2O2 and cisplatin (19, 20). In addition, protective effects of Lcn2 on ROS mediated oxidative damages following irradiation have been reported previously. Hence, it seems that Lcn2 acts as a protective protein against damages caused by chemotherapeutics induced-oxidative stress (21). Considering broad application of cisplatin in treatment of various types of cancer, and the fact that it exerts its cytotoxic effects via genotoxic activity, in the present study, we evaluated if Lcn2 overexpression has cytoprotecitve effect on a normal human cell line, and if the probable cytoprotective effect is mediated via protection of cellular genome. 

## Experimental

*Cell culture*

HEK293 cell line was obtained from National Cell Bank of Iran (Pasteur Institute of Iran, Tehran, Iran). The cells were grown in RPMI-1640 medium (Gibco-BRL, Germany) containing 10% fetal bovine serum (Gibco-BRL, Germany), 100 U/mL penicillin, and 100 μg/mL streptomycin at 37 °C and in the presence of 5% CO2.

*Cell transfection and selection of stable cells*

The recombinant plasmid pcDNA3.1-Lcn2 was constructed previously (20) and used for transfection of 3.0**-**3.5 × 10^4^ HEK293 cells in well of a 24-well plate by X-tremeGENE (Roche, Germany) transfection reagent as instructed by the manufacturer and also as described elsewhere (22). Furthermore, as control, HEK293 cells were transfected whit non-recombinant pcDNA3.1 plasmid. In order to obtain cells stably expressing Lcn2, forty-eight hours post transfection, the transfected HEK293 cells were exposed to 400 (µg/mL) Geneticin (Sigma-Aldrich, Germany) for at least 3 weeks. Then, several stable colonies were picked up and transferred to wells of a 96-well plate and the cells were propagated stepwise to 6-well plates and finally to cell culture flasks. Expression of Lcn2 mRNA and protein were evaluated by RT-PCR and ELISA methods and the obtained Lcn2 over-expressing cells were designated as Lcn2-HEK293 cell line. 

*Evaluation of Lcn2 mRNA over-expression by RT-PCR*

RNA extraction and RT-PCR analysis was performed as described earlier (23). Briefly, total RNA from stable cells was isolated with RNeasy MiniPrep kit (Qiagen, Germany) according to the manufacturer’s protocol. In order to eliminate possible contamination with genomic DNA, 500 ng of extracted total RNA was treated by DNAseI (Thermoscientific, USA) followed by heat inactivation, andreverse transcription was performed by RevertAid™ First Strand cDNA Synthesis kit (Thermoscientific, USA) according to the manufacturer’s protocol. PCR was performed using Taq DNA polymerase (Cinnagen, Iran). For amplification of the Lcn2 cDNA, primer pair LCNFR and LCNRV were used (Table 1) which amplifies a fragment of about 240 bp. Amplification of GAPDH cDNA was performed using GAPDHFR and GAPDHRV primers (Table 1) and considered for normalization. The PCR condition included an initial denaturation step at 94 °C for 5 min, followed by 30 cycles of denaturation at 95 °C for 45 sec, annealing at 55 °C for 90 sec, and extension at 72 °C for 45 sec, and a final extension step at 72 °C for 5 min. Finally, the RT-PCR products were analyzed on 1% agarose gel.

*ELISA*

Culture medium of stable HEK293 cells was 20 times diluted and used for assessment of Lcn2 protein expression using human Lipocalin2/NGAL Quantikine ELISA Kit (R&D system, USA) according to the manufacturer’s protocol and as described before (24). Briefly, 100 µL of assay diluent were added to each well of the ELISA strips. Then, 50 µL of Standard, control, or samples were added to each well, and the strips were covered with a plate sealer, and incubated at 2-8 °C for 2 h. Afterwards, the content of each well was aspirated and washed 4 times. Then, 200 µL of cold conjugate was added to each well and following sealing the strips with a new plate sealer, they were incubated at 2-8 °C for 2 h. Next, the content was aspirated and wells were washed 4 times, followed by addition of 200 µL Substrate Solution to each well. Following 30 min incubation at room temperature and in a dark place, 50 µL stop solution was added to each well and the absorbance was read at 450 nm (using 540 nm as wavelength correction) (Bio-TEK, USA). Finally, the concentration of Lcn2 was determined according to the absorbance of standard samples provided by the kit.

*Cytotoxicity assays*

The cytotoxic effect of cisplatin, on Lcn2-HEK293 cell line was determined by MTT assay (25). To do this, 5 × 10^3^ cells were seeded in each well of a 96-well plate and incubated under mentioned cell culture condition for 24 h. Afterwards, different concentrations of cisplatin were added to the wells to obtain final concentrations of 0.1-10 μg/mL. After 48 h of further incubation, MTT solution (5 mg/mL) (MTT, Sigma, Germany) was added to each well at a final concentration of 0.5 mg/mL and plates were further incubated at 37 ºC in 5% CO2 atmosphere for 4 h. Finally, the medium was removed and 150 μL DMSO was added to each well to dissolve formazan crystals, and absorbance was read at 570 nm.

*Evaluation of protective effect of Lcn2 on cisplatin using alkaline comet assay*

In order to evaluate the protective effect of Lcn2 overexpression on cisplatin genotoxicity by alkaline comet assay was performed as described before (26, 27). In this regard, 5 × 10 cells were seeded per well in a 12-well plate in RPMI-1640 supplemented with 10% FBS, 100 mg/mL penicillin and 100 mg/mL streptomycin. After 24 h, the medium was replaced with RPMI-1460 containing 1 or 2 µg/mL cisplatin and the plate was incubated at 37 °C for 1 h. Then, the cells were trypsinised and used in comet assay for evaluation of DNA damage.

For comet assay, first, some slides were coated with 1% NMP (normal melting agarose) (Cinnagen, Iran) and allowed to dry for 1 day at room temperature. Then, the treated cells were mixed with 1 mL of 1% LMP (low melting agarose) (Sigma, USA) and placed on the pre-coated slides, covered by cover slip and kept at 4 °C for 10 min. Then, the coverslip were removed and Lysis buffer (2.5 M NaCl,100 mM EDTA, 0.2 M NaOH, 10 mMTris, 1% Triton X-100, pH 10) were added to the slides and they were kept at 4 °C for 40 min. Then, the slides were rinsed with deionized water and submerged in electrophoresis buffer (0.3 M NaOH, 1 mM EDTA, pH > 13) for 40 min. Then, electrophoresis was performed with the same buffer at 300 mA and 25 V for 40 min. Next, the slides were removed and submerged in neutralizing buffer for 10 min. 

Finally, the slides were stained with ethidium bromide (20 μg/mL, Sigma-Aldrich, USA) for 5 min and rinsed with deionized water. Then, the slides were dried on an arid surface and observed under ×400 magnification fluorescent microscope (CETI, England) with an excitation filter of 510-560 nm and a barrier filter of 590 nm and the photos of these slides were taken. For each analysis at least 100 cells were needed to be present in each captured photo. DNA damage was measured using the comet score software and expressed as (comet length, % DNA in Tail and Tail Moment), and results were given as mean ± SEM. 

*Statistical analysis*

The results are expressed as mean ± SEM of three independent experiments. Differences between groups were compared using ANOVA with Tukey Multiple Comparison Test as a post-hoc test. Statistical analysis was accomplished by IBM-SPSS software v21, (IBM Analytics, USA).

## Results

*Evaluation of Lcn2 over-expression in the LCN2-HEK293 cells*

**Figure 1:**
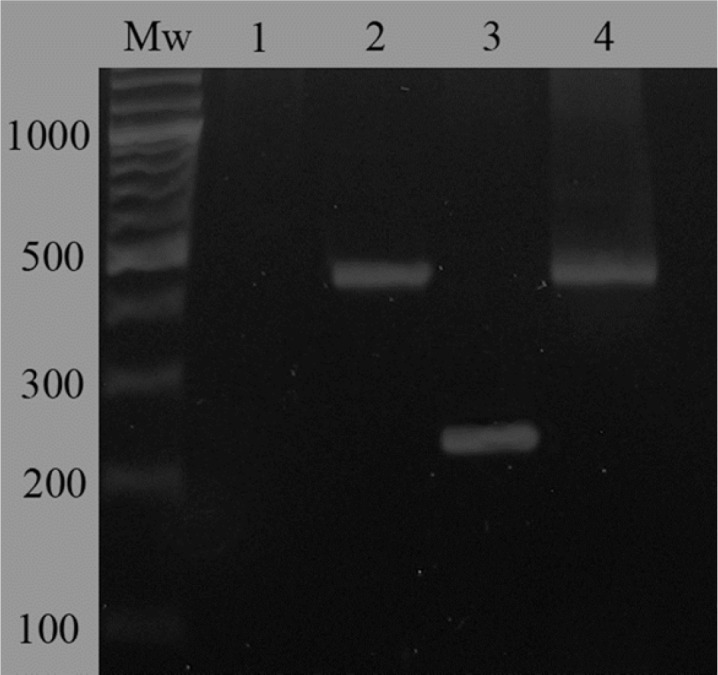
Evaluation of mRNA expression of Lcn2 gene in Lcn2-HEK293 cells comparing to mock-transfected HEK293 cells by RT-PCR. A 240-bp fragment indicated over-expression of Lcn2 in Lcn2-HEK293 cells (lane 3). Not any amplified band was observed in case of the mock-transfected cells confirming the very low level of Lcn2 expression in these cells (lane 1). Evaluation of GAPDH mRNA expression in mock-transfected and Lcn2-HEK293 cells was considered for normalization (lanes 2 and 4, respectively). Mw corresponds to molecular weight marker

For evaluation of Lcn2 over-expression by the transfected cells, RT-PCR and ELISA were performed. In case of the RT-PCR analysis, a band of about 240 bp indicating the over-expression of Lcn2 mRNA by the Lcn2-HEK293 cells was observed (Figure 1, lane 3). However, in case of the cells transfected with non-recombinant pCDNA3.1 not any amplification was detected. In addition, amplification of GAPDH fragment was performed as a positive control and for normalization (Figure 1, lanes 2 and 4).

*Over-expression of Lcn2 decreased cisplatin toxicity*

**Figure 2:**
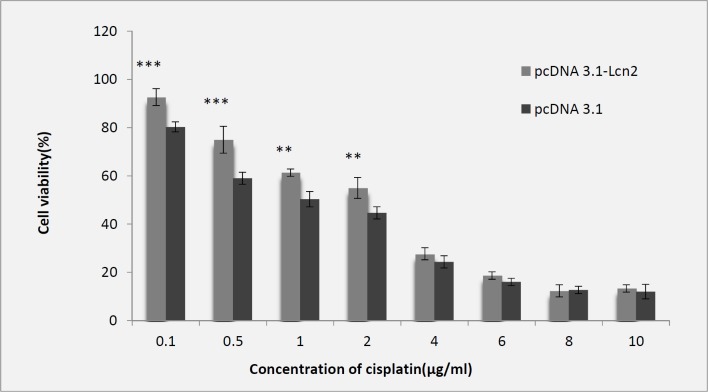
Evaluation of cytotoxic effect of cisplatin on Lcn2-HEK293 cells comparing to the control cells (transfected with empty pCDNA3.1 plasmid) by MTT assay (IC50 = 2 µg/mL) (Mean ± SD,

For evaluation of cisplatin toxicity following over-expression of Lcn2, the Lcn2-HEK293 cells were exposed to different concentrations of cisplatin including 0.1 to 10 (µg/mL), which showed higher cell viability at lower concentrations (0.1 and 2 (µg/mL)) of cisplatin comparing to the control cells transfected with the empty pCDNA3.1 plasmid. However, at cisplatin concentrations higher than 2 µg/mL not any significant difference between viability of the Lcn2-HEK293 or mock HEK293 cells was observed (Figure 2).

*Protective effect of Lcn2 over-expressionon cisplatin induced DNA damage*

**Figure 3:**
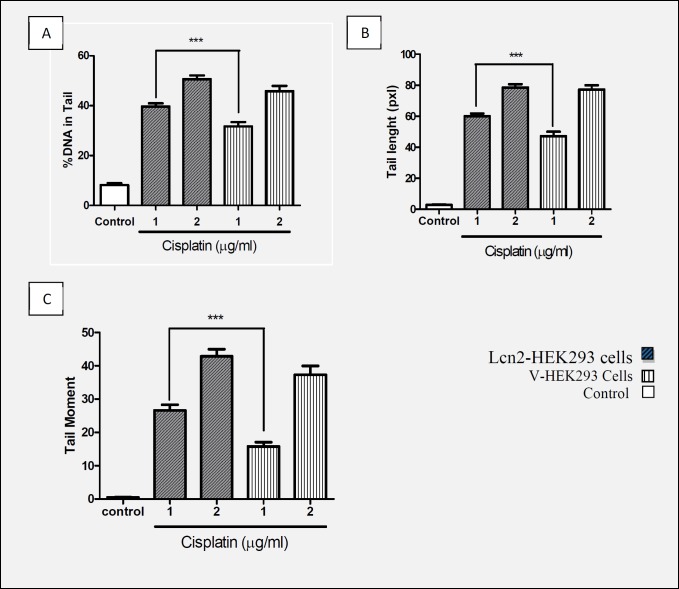
Comparison of (A) % of DNA in Tail, (B) Tail length, and (C) Tail moment with concentrations of 1 and 2 (µg/mL) of cisplatin in Lcn2-HEK293 cells and HEK293-V cells that indicated protective effect Lcn2 against genotoxicity cisplatin (Mean ± SEM ***: *P* < 0.001).

The Lcn2-HEK293 cells and mock transfected HEK293 cells (as control) were treated with 1 and 2 µg/mL cisplatin for 1 h, then subjected to alkaline comet assay as described above. The results indicated lower extent of DNA lesion in the Lcn2-HEK293 cells in case of exposure to 1 (µg/mL) cisplatin. However, in case of exposure of the cells to 2 µg/mL cisplatin not any significant differences were observed (Figure 3).

## Discussion

Cisplatin is one of the most potent anti-tumor chemicals, so it is widely used to treat many types of cancers (28, 29). This drug conducts its effects via accumulation of platinum in tissues followed by triggering oxidative stress (9). It is presumed that DNA is the first biological target of cisplatin (30-32). In addition, it has been proved that cisplatin and carboplatins are the first to initiate chromosome damages in micronucleus test systems (33, 34). Cell DNA is continuously under pressure with oxidative stress by endogenous and exogenous sources such as metabolism, inflammation, ionizing radiations, and other various chemicals (35).

**Table 1:** The sequences of primer pairs used in RT-PCR analysis of mRNA expression

**Primer Name**	**Primer sequences**
Lcn2Fr	5’-TCACCTCCGTCCTGTTTAGG-3’
Lcn2Rv	5’-CGAAGTCAGCTCCTTGGTTC-3’
GAPDHFR	5’-GATGGCCCCTCCGGGAAA-3’
GAPDHRV	5’-AGGGGTCTTACTCCTTGGA -3’

**: *P* < 0.01, ***: *P *< 0.001).

In order to protect cells against oxidative damages of DNA, there are some DNA glycosylases in cells which identify and remove damaged bases (36, 37). However, there might be other cell protective factors which maintain genome integrity and fidelity. Lcn2 is a cytoprotective protein which its important cytoprotective roles in various damaging conditions especially under oxidative stress conditions have been reported (24, 38). Therefore, the aim of the present study was to evaluate the cytoprotective effect of Lcn2 in a normal human cell line following cisplatin treatment, and also to check if its cytoprotective effect is mediated via prevention of genotoxicity. In this regard, HEK293 cells were transfected with a plasmid containing Lcn2 expression cassette and genomic integration of the cassette was induced by antibiotic screening of the transfected cells. Afterwards, overexpression of Lcn2 comparing to non-transfected cells was confirmed by RT-PCR and ELISA techniques. Then, the cells overexpressing Lcn2 and also normal cells were exposed to various concentration of cisplatin and subjected to MTT assay searching for any cytoprotective effects. Finally, the cells were subjected to comet assay for evaluation of genoprotective effect of lipocalin following cisplatin treatment.

Many studies have reported increased Lcn2 concentration following production and accumulation of free radicals in cells which proves Lcn2 effects against ROS mediated oxidative stresses. In addition, another study showed increased Lcn2 expression due to the stress caused by severe hypoxia and ischemic heart disease (39). Researches also showed protective effect of Lcn2 against the toxicity caused by H2O2 and cisplatin on CHO cells (18, 19). Also, in the present study, we observed that up-regulation of Lcn2 expression makes HEK293 cells more resistant to cytotoxicity induced by cisplatin concentration of 0.1 to 2 µg/mL. However, no significant difference with the control cells was observed when the cells were treated with cisplatin concentrations higher than 2 µg/mL. Having confirmed the protective effect of Lcn2 overexpression on the viability of the recombinant cells against lower concentrations of cisplatin, and considering the aforementioned results of other studies noticing that cisplatin imposes its toxicity to cells through oxidative damages to genome, and that the increase in amount of Lcn2 in oxidative stress condition has been frequently reported, we evaluated if the mechanism of the observed protection is via geno-protective effects. This was assayed by comet assay, as a simple, sensitive and standard method to measure DNA damages (40, 41). Therefore, following cisplatin treatment of the control and Lcn2 overexpressing cells with the concentrations of 1 and 2 µg/mL, the cells were subjected to alkaline comet assay. As it is shown, there was a significant difference between the amount of tail length, % of DNA in tail, and tail moment when the Lcn2 overexpressing cells were exposed to cisplatin concentration of 1 µg/mL, comparing to the mock transfected cells. However, at higher cisplating concentration, *i.e.* 2 µg/mL, there was no significant difference between the three evaluated parameters comparing to the mock transfected cells. This confirmed that in addition to other mechanisms suggested for cytoprotective effects of Lcn2, genoprotectivity might be also an effective mechanism of this protein. However, since lipocalin 2 over expression was unable to protect genomic DNA against oxidative damages at cisplatin concentrations higher than 2 µg/mL, which indicates limited cytoprotective effects of this protein.

In conclusion, in the present study we showed that geno-protectivity is one of the mechanisms implicated in cytoprotective effects of Lcn2. However, additional studies to further identifying detailed mechanisms of this genoprotective effect must be performed.
